# Rac and Arp2/3-Nucleated Actin Networks Antagonize Rho During Mitotic and Meiotic Cleavages

**DOI:** 10.3389/fcell.2020.591141

**Published:** 2020-11-17

**Authors:** Debadrita Pal, Andrea Ellis, Silvia P. Sepúlveda-Ramírez, Torey Salgado, Isabella Terrazas, Gabriela Reyes, Richard De La Rosa, John H. Henson, Charles B. Shuster

**Affiliations:** ^1^Department of Biology, New Mexico State University, Las Cruces, NM, United States; ^2^Department of Biology, Dickinson College, Carlisle, PA, United States

**Keywords:** cytokinesis, Rac, Rho, Arp2/3, meiosis, sea urchin, sea star, contractile ring

## Abstract

In motile cells, the activities of the different Rho family GTPases are spatially segregated within the cell, and during cytokinesis there is evidence that this may also be the case. But while Rho’s role as the central organizer for contractile ring assembly is well established, the role of Rac and the branched actin networks it promotes is less well understood. To characterize the contributions of these proteins during cytokinesis, we manipulated Rac and Arp2/3 activity during mitosis and meiosis in sea urchin embryos and sea star oocytes. While neither Rac nor Arp2/3 were essential for early embryonic divisions, loss of either Rac or Arp2/3 activity resulted in polar body defects. Expression of activated Rac resulted in cytokinesis failure as early as the first division, and in oocytes, activated Rac suppressed both the Rho wave that traverses the oocyte prior to polar body extrusion as well as polar body formation itself. However, the inhibitory effect of Rac on cytokinesis, polar body formation and the Rho wave could be suppressed by effector-binding mutations or direct inhibition of Arp2/3. Together, these results suggest that Rac- and Arp2/3 mediated actin networks may directly antagonize Rho signaling, thus providing a potential mechanism to explain why Arp2/3-nucleated branched actin networks must be suppressed at the cell equator for successful cytokinesis.

## Introduction

Cytokinesis is the final phase of cell division where in animal and fungal cells, a transient assemblage of actin and myosin II assembles at the former metaphase plate to effect the partitioning of the two daughter cells. Due to the unique spatiotemporal parameters that drive contractile ring assembly, cytokinesis is often considered distinct from other motility events in animal cells, yet parallels exist between cytokinesis and polarized cell migration, especially in regard to the Rho family GTPases. In crawling cells, there is a symmetry-breaking event that drives the localized activation of the small GTPases Rac and/or Cdc42, which in turn promote the elaboration of viscoelastic, branched actin networks at the leading edge to effect forward protrusion (Blanchoin et al., 2014; Schaks et al., 2019). In contrast, Rho activity at the rear promotes contractility and retraction of the trailing edge (Machacek et al., 2009). During cytokinesis, there is an analogous spatial segregation of Rho GTPase activity, with Rho activated at the cell equator (Yoshizaki et al., 2003; Bement et al., 2005), whereas Rac/Cdc42 activity is cleared from the cell equator and enriched in domains of cytoplasm undergoing post-mitotic spreading (Yoshizaki et al., 2003). There are multiple potential mechanisms by which Rho and Rac may antagonize one another (Guilluy et al., 2011), and there is evidence that mutual Rho-Rac antagonism may be a fundamental aspect of polarized cell migration (Byrne et al., 2016). Whether a Rho-Rac negative regulatory circuit plays a functional role during cytokinesis has not been firmly established.

Following anaphase onset and the initiation of mitotic exit, symmetry is broken by the mitotic apparatus, which specifies the equatorial position of Rho activation and contractile ring assembly (Basant and Glotzer, 2018). The primary component of the signaling apparatus that determines the cleavage plane is the centralspindlin complex, comprised of the Mitotic Kinesin Like Protein (MKLP1) and Cyk4/MgcRacGAP, which not only plays a role in organizing the post-anaphase central spindle (Mishima et al., 2002) but also recruits the RhoGEF Ect2 (Yüce et al., 2005) to activate Rho at the cell equator. The precise role of GAP activity of Cyk4 has been difficult to elucidate, and there have been multiple proposed roles for Cyk4 that invoke RhoGAP activity, RacGAP activity, or indirect promotion of Rho activation (Canman et al., 2008; Miller et al., 2008; Loria et al., 2012; Tse et al., 2012; Breznau et al., 2015; Zhang and Glotzer, 2015; Zhuravlev et al., 2017). The notion of a negative regulatory role for Rac has been supported by studies in cultured mammalian cells (Yoshizaki et al., 2004; Bastos et al., 2012; Cannet et al., 2014), *Drosophila* (D’Avino et al., 2004) and *Caenorhabditis elegans* embryos (Canman et al., 2008; Zhuravlev et al., 2017). There are multiple effector pathways by which activated Rac might antagonize cytokinesis (Jordan and Canman, 2012), and there is evidence that Rac may disrupt cytokinesis through PAK-mediated promotion of cell adhesion (Bastos et al., 2012) or through WAVE-mediated activation of Arp2/3 (Canman et al., 2008; Cannet et al., 2014). While these studies implicate Rac as a negative regulator of cytokinesis, a common mechanism by which Rac antagonizes Rho-mediated contractile ring assembly yet to emerge.

To further explore the notion that the spatiotemporal regulation of cytokinesis might involve antagonism between Rac and Rho, we modulated Rac activity during mitotic divisions in early sea urchin embryos and meiotic divisions in sea star oocytes. In contrast to symmetrical divisions, polar body formation exemplifies a highly asymmetric division that involves a (1) surface contraction wave (SCW) that terminates at the site of polar body formation, (2) protrusion of the anaphase spindle and (3) centralspindlin-mediated ring constriction (Zhang et al., 2008; Maddox et al., 2012; Satoh et al., 2013), and both Rac and Arp2/3 have been directly or indirectly implicated in this process (Maddox et al., 2012). Live cell imaging of sea urchin zygotes revealed that while neither Rac nor Arp2/3 were not required for cell division in the early embryo, expression of activated mutants of Rac disrupted both cytokinesis and normal actin dynamics throughout the embryo. However, the effect of dysregulated Rac activity could be suppressed by effector-binding mutations or direct inhibition of Arp2/3-mediated actin polymerization. During oocyte maturation, Rac and Arp2/3 were required for spindle docking at the cortex, but dysregulated Rac activity suppressed polar body formation in an Arp2/3-dependent manner by directly suppressing the wave of Rho activity that drives the SCW during anaphase I. Together, our results suggest that Rac-stimulated branched actin networks may act as a direct antagonist to Rho activity, which may provide an additional layer of spatial regulation to promote efficient daughter cell partitioning during cytokinesis.

## Results

### Actin Organization in the Sea Urchin Zygote

**FIGURE 1:**
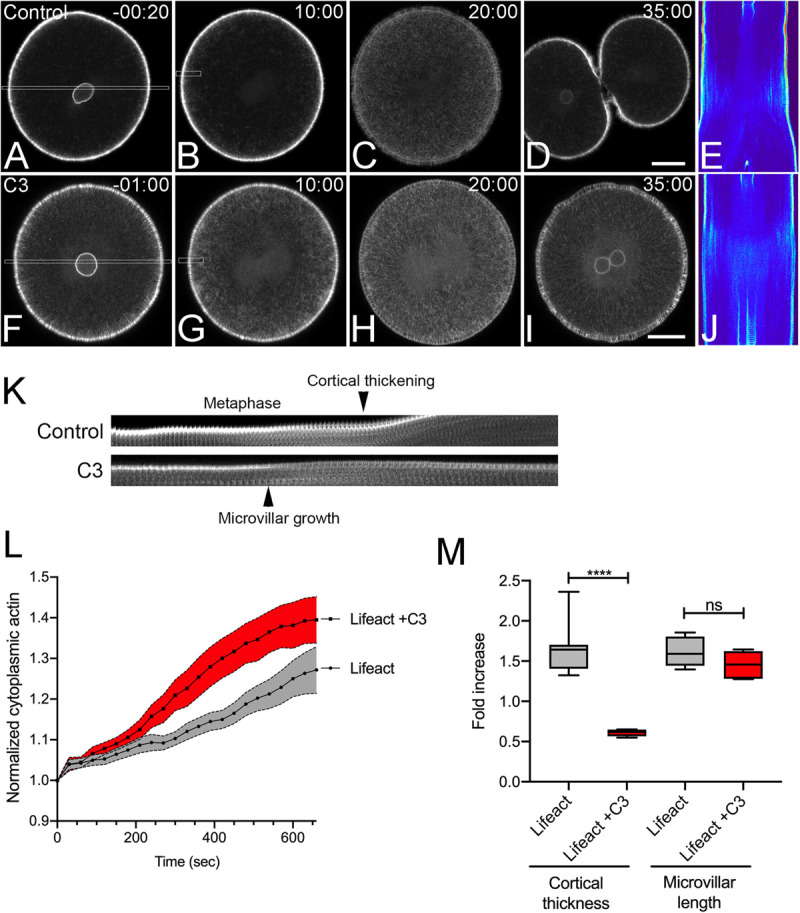
Actin dynamics in the dividing sea urchin embryo. Frames from time-lapse movies of *L. variegatus* embryos injected with either recombinant Lifeact-GFP (**A–E**, final intracellular concentration of 0.21 μM) or Lifeact-GFP and C3 Transferase (**F–J**, final intracellular concentration 0.27 μM). Bar, 30 μm. Time points denote minutes post-nuclear envelope breakdown (NEB). **(E,J)** Corresponding kymographs of sections taken through the long axis of the spindle as denoted by the rectangles in panels **(A,F)**. Prior to nuclear envelope breakdown a bright rim of perinuclear actin is visible **(A,F)**. Upon mitotic entry, microvilli and their rootlets shorten, and there is a gradual increase in deep cytoplasmic actin initiating at the cortex and accumulating inward up until anaphase onset **(C,E,H,J)**. **(K)** Kymograph of cortical microvillar dynamics in the polar regions of control- and C3-injected embryos, taken from regions denoted by the small rectangles in Panels **(B,G)**. **(L)** Quantification of cytoplasmic actin levels beginning 1 min post-NEB up until the metaphase-anaphase transition (Mean ± SEM, *n* = 6 cells per condition). While there was not a qualitative effect of Rho inactivation on cytoplasmic actin, there was a significant increase in cytoplasmic Lifeact fluorescence in C3-injected embryos. **(M)** Quantitation of the increases in cortical thickness and microvillar length that accompanies the metaphase-anaphase transition in control and C3-injected embryos (*n* = 6 cells per condition, whiskers denote minimum and maximum values), where a value of 1.0 represents length or thickness 1 min prior to the metaphase-anaphase transition. While there was no significant difference in the growth of microvilli, Rho inactivation blocked the thickening of actin cortex. ****p < 0.0001.

To better understand the respective roles of Rho and Rac in regulating cell shape change and cytokinesis, we first employed live cell imaging of actin during the first division of the sea urchin embryo to establish a baseline by which further perturbations could be compared (Figure 1). Injection of recombinant Lifeact-GFP labeled actin filaments throughout the cortex and cytoplasm without compromising cell viability, with embryos surviving through gastrulation. During interphase, there was a bright, perinuclear rim of EGFP-Lifeact that increased in intensity until nuclear envelope breakdown (Figures 1A,F). Lifeact labeled the dense array of microvilli and microvillar rootlets that extended into the cytoplasm. Following nuclear envelope breakdown, the rootlets retracted and microvilli remained short and dense (Figures 1A,B) up until the metaphase-anaphase transition, at which time microvilli elongated (Figures 1C,K,M), consistent with previous observations made in fixed specimens (Wong et al., 1997). Submembranous cortical actin, obscured by microvilli earlier in mitosis, could be observed undergoing a transient thinning at the metaphase-anaphase transition, followed by an isotropic thickening just prior to the onset of cytokinesis (Figures 1C,K,M and Supplementary Movie S1). There was also an elaboration of actin filaments in the cytoplasm that peaked after anaphase onset and dissipated during cytokinesis, which appeared in kymographs as parallel streaks (Figures 1D,E,L and Supplementary Movie S1). Lastly, inactivation of Rho with C3 transferase blocked cytokinesis (Figures 1F−K), but had no overt qualitative effects on the different actin populations. However, Rho inactivation did result in an increase in the accumulation of cytoplasmic actin (Figure 1L) and disrupted the thickening of the cortex during mitotic exit (Figures 1H−K,M and Supplementary Movie S2). Thus, of all the different actin populations identified by live cell imaging, Rho appeared to be only required for the thickening of the cell cortex during mitotic exit as well as its well-characterized role in contractile ring formation (Mabuchi et al., 1993).

### Inactivation of Rac Is Required for Cytokinesis

**FIGURE 2:**
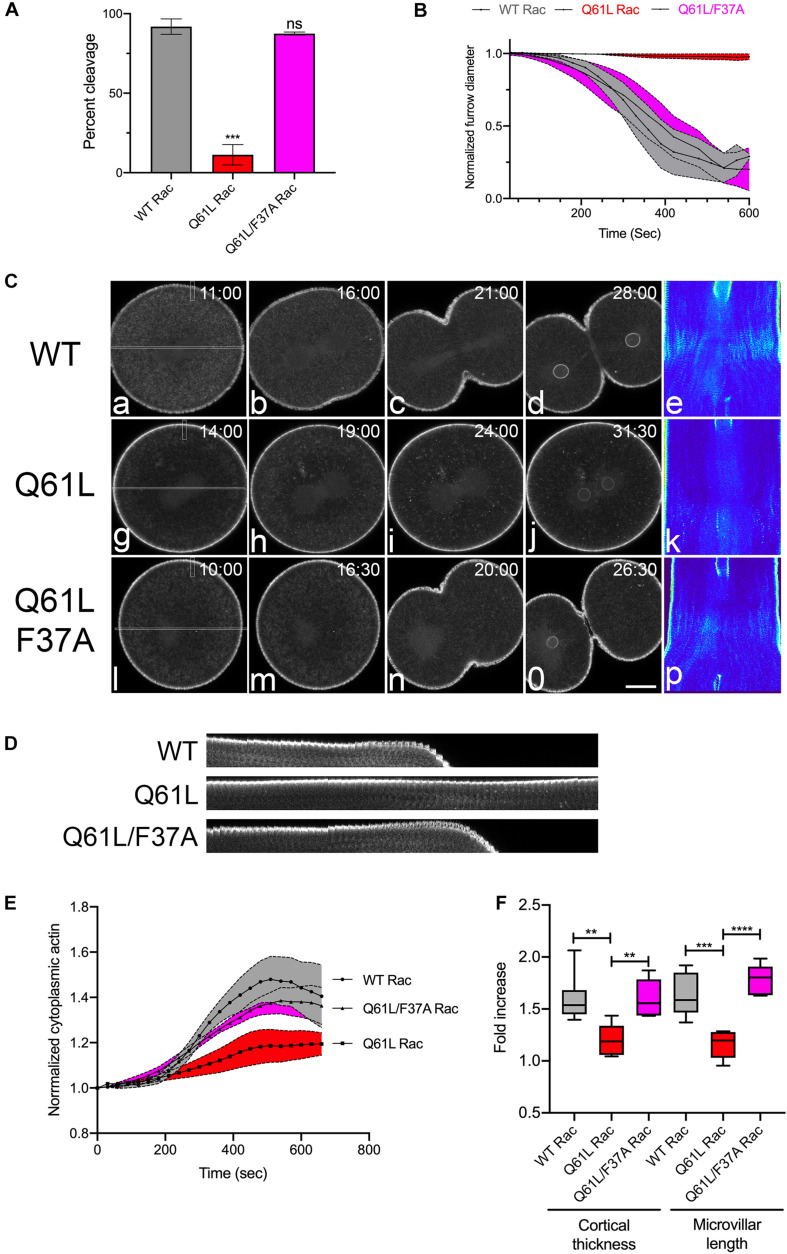
Activated Rac suppresses actin dynamics and cytokinesis. **(A)** Cleavage rates for *L. variegatus* embryos injected with WT, Q61L or Q61L/F37A Rac mRNA (final intracellular concentration 2.5 μg/ml) and recombinant Lifeact-GFP (final intracellular concentration 0.2 μM) immediately after fertilization and cultured at 20°C until un-injected controls completed cytokinesis. Mean ± SEM for three experimental replicates, with >21 cells per experiment. ***p < 0.001. **(B)** Furrow ingression profiles for injected cells, beginning at the initiation of furrowing (mean ± SEM, *n* = 5 per condition). **(C)** Frames from time-lapse movies of *L. variegatus* embryos injected with mRNA encoding WT or mutant Rac. Time points denote minutes post-NEB. Bar, 30 μm. Rectangles in panels **(a,g,l)** denote the region used to generate the kymographs in panels **(e,k,p)**. Small rectangles denote regions used to generate the kymographs in panel **(D)**. Bar, 30 μm. **(D)** Kymograph highlighting microvillar growth and cortical actin dynamics during mitotic exit in WT or mutant Rac-expressing cells. **(E)** Quantification of cytoplasmic actin levels beginning 1 min post-NEB up until the metaphase-anaphase transition (Mean ± SEM, *n* = 6 cells per condition). Activated Rac (Q61L, red) suppressed the increase in cytoplasmic actin in comparison to WT (gray), whereas activated Rac containing the effector-binding mutation F37A (Q61L/F37A, magenta) had no effect on cytoplasmic actin levels. **(F)** Quantitation of the increases in cortical thickness and microvillar length that accompanies the metaphase-anaphase transition in embryos expressing WT and mutant Rac (*n* = 6 cells per condition, whiskers denote minimum and maximum values), where a value of 1.0 represents length or thickness 1 min prior to the metaphase-anaphase transition. Activated Rac blocked both cortical thickening and microvillar elongation, but these actin dynamics were rescued in embryos expressing the double mutant that is incapable of promoting WAVE-mediated Arp2/3 activation. **p < 0.005, ****p < 0.0001.

In the sea urchin zygote, Rac transcript levels are seven-fold higher than Cdc42 (Tu et al., 2014) and given the proposed antagonistic role of Rac during cytokinesis (Canman et al., 2008), efforts were focused on Rac. Microinjection of mRNA encoding wild type or dominant-negative (T17N) Rac together with EGFP-Lifeact into sea urchin embryos resulted in morphologically indistinguishable mesenchymal blastulae (Supplementary Figure S1B), suggesting that Rac was dispensable for at least the first ten cleavages. To further explore the notion that Rac may play an antagonistic role in cytokinesis, a constitutively active (Q61L) form of sea urchin Rac was generated as well as an effector-binding mutant that blocked interaction with the WAVE complex (F37A) and thus the downstream activation of Arp2/3 (Lamarche et al., 1996). The activities of these mutants were validated in cultured cells (Supplementary Figure S1C) and then expressed in *Lytechinus variegatus* embryos during the first cell cycle (Figure 2). In contrast to wild-type (WT) Rac (Figures 2A−C and Supplementary Movie S3), Q61L Rac had a strong negative effect on cytokinesis at the first division, with Q61L Rac expressing cells failing to initiate furrowing (Figure 2B, Supplementary Movie S3). However, the effector-binding F37A mutation suppressed the inhibitory effect of constitutively active Rac, both in terms of the overall success rates of cleavage (Figure 2A) as well as the rate of furrow ingression where WT and Q61L/F37A expressing embryos had superimposable furrowing rates (Figure 2B). Examination of actin dynamics in Q61L Rac embryos revealed no gross disruptions in actin organization during the first cell cycle (Figure 2C and Supplementary Movie S3), although in later divisions, actin organization was more dramatically altered, with pseudopodial processes extending and retracting from the cell surface (Supplementary Movie S4). Quantification of actin dynamics revealed that the accumulation of cytoplasmic actin, thickening of the actin cortex and microvillar elongation were all significantly disrupted in Q61L-expressing embryos, but suppressed in embryos expressing Q61L/F37A Rac (Figures 2D−F and Supplementary Movie S3). Thus, in addition to the two Rho-independent actin populations (microvillar and cytoplasmic actin), dysregulated Rac significantly suppressed Rho-dependent cortical thickening and contractile ring formation, and this effect appeared to be dependent on effectors that were sensitive to the F37A mutation.

### Direct Inhibition of the Arp2/3 Complex Rescues Furrowing in Q61L-Expressing Cells

**FIGURE 3:**
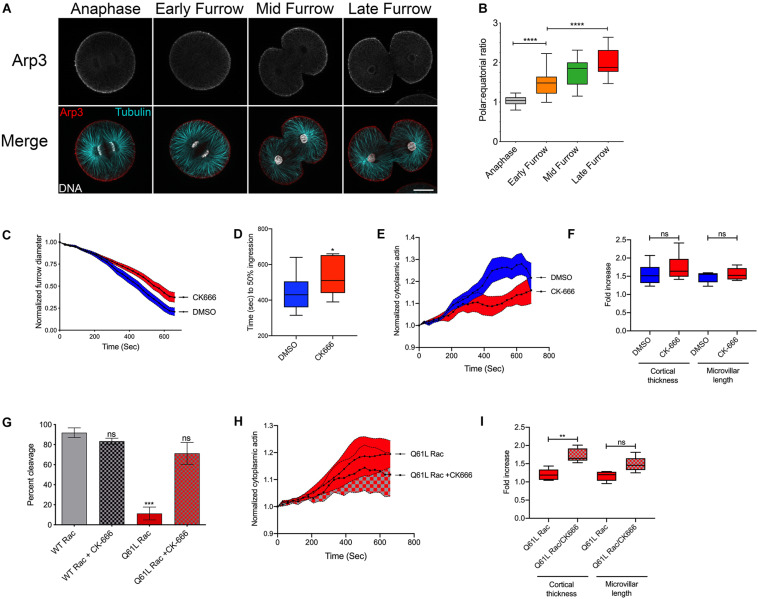
Activated Rac suppresses cytokinesis through Arp2/3. **(A,B)** *S. purpuratus* embryos probed for Arp3 (top row in grayscale, red in the merged image), tubulin (cyan), and DNA (White). Bar, 30 μm. As cytokinesis progresses, Arp2/3 is increasingly excluded from the furrow region. ****p < 0.0001. **(C)** Arp2/3 delays but does not block cytokinesis. Embryos were treated with 0.1% DMSO or 100 μM CK-666 prior to nuclear envelope breakdown, and furrow diameter was measured by Nomarski/DIC starting at the initiation of furrowing (mean ± SEM, *n* = 13 for DMSO, *n* = 12 for CK-666). **(D)** Comparison of the rates of furrowing (time to 50% ingression), *p < 0.05. **(E)** Quantification of cytoplasmic actin levels in either DMSO- or CK-666-treated embryos beginning 1 min post-NEB up until the metaphase-anaphase transition (Mean ± SEM, *n* = 6 cells per condition). **(F)** Quantitation of the increases in cortical thickness and microvillar length that accompanies the metaphase-anaphase transition in DMSO or CK-666-treated embryos expressing (*n* = 6 cells per condition, whiskers denote minimum and maximum values), where a value of 1.0 represents length or thickness 1 min prior to the metaphase-anaphase transition. No significant differences were detected for either actin population. **(G)** Arp2/3 inhibition rescues cytokinesis in embryos expressing activated Rac. Q61L Rac data replicated from Figure 2A. Mean ± SEM for three experimental replicates, >24 cells per experiment. ***p < 0.001. **(H)** Cytoplasmic actin levels in embryos expressing Q61L Rac in the absence or presence of CK-666, beginning 1 min post-NEB up until the metaphase-anaphase transition (Mean ± SEM, *n* = 6 cells per condition). **(I)** Quantitation of the increases in cortical thickness and microvillar length that accompanies the metaphase-anaphase transition in embryos expressing Q61L Rac in the absence or presence of CK-666 (*n* = 6 cells per condition, whiskers denote minimum and maximum values), where a value of 1.0 represents length or thickness 1 min prior to the metaphase-anaphase transition. **p = 0.005.

Constitutively active Rac blocked cytokinesis through effectors sensitive to the F37A mutation (Figure 2), which has been previously reported to affect WAVE activation and Arp2/3-mediated actin polymerization (Lamarche et al., 1996). Indeed, immuno-labeling of Arp3 in *Strongylocentrotus purpuratus* embryos revealed that Arp2/3 is isotropically distributed throughout the polar and equatorial cortices of anaphase embryos, but as cytokinesis progressed, Arp2/3 was increasingly cleared from the equatorial plane (Figures 3A,B). Pharmaceutical inhibition of Arp2/3 with the small molecule inhibitor CK-666 (Nolen et al., 2009) did not inhibit cytokinesis during the first cleavage, but did slow the rate of furrowing (Figure 3C) such that the time required to reach 50% ingression was increased by approximately 90 s (Figure 3D). Quantification of the different actin populations revealed that while a depression of cytoplasmic actin could be observed in CK-666 treated embryos (Figure 3E), there were no significant differences in the increases in cortical thickness and microvillar length in Arp2/3-inhibited embryos (Figure 3F).

Introduction of the F37A mutation neutralized the ability of constitutively active Rac to block cytokinesis (Figure 2). To further explore the potential role of Arp2/3, embryos expressing WT or Q61L Rac were scored for cleavage in the absence or presence of the Arp2/3 inhibitor. Direct inhibition of the Arp2/3 complex rescued cytokinesis in Q61L Rac-expressing embryos (Figure 3G). And while CK-666 did not affect the dynamics of cytoplasmic actin in Q61L Rac embryos (Figure 3H), Arp2/3 inhibition did rescue Rho-dependent thickening of the cortical layer (Figure 3I). Thus, while Rac is capable of activating a variety of downstream effector pathways (Jordan and Canman, 2012), Rac’s promotion of Arp2/3-mediated branched actin networks appeared to be what was responsible for Rac’s deleterious effects on cytokinesis.

### Rac and Arp2/3 Involvement in Polar Body Formation in Starfish Oocytes

**FIGURE 4:**
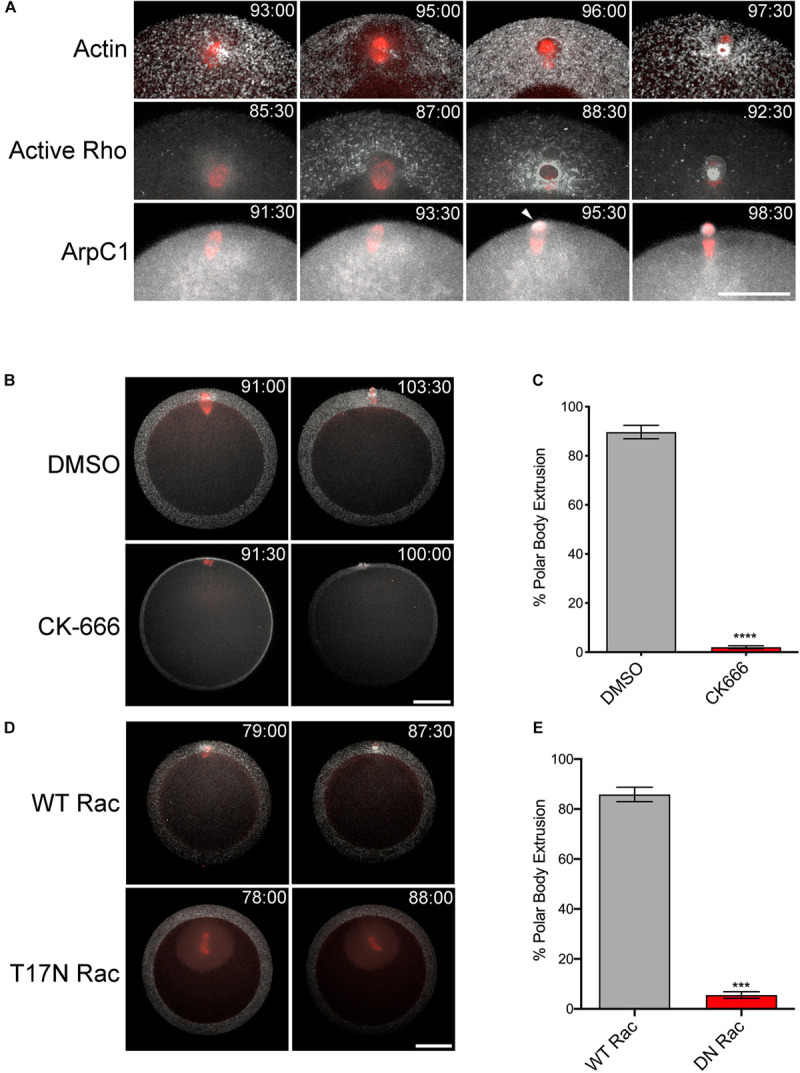
Rac and Arp2/3 are required for polar body extrusion in sea star oocytes. **(A)** First polar body extrusion in *P. miniata* oocytes co-expressing Lifeact (white) and GFP-tubulin (red), rGBD-GFP (white) and mCherry-EMTB (red), and ArpC-GFP (white) and mCherry-EMTB (red). Time points indicate minutes post-hormone activation. Arrow denotes protruding polar body. Bar, 50 μm. **(B)** *P. miniata* oocytes co-expressing Lifeact (white) and GFP-tubulin (red) were activated with maturation hormone and 40 min following germinal vesicle breakdown, were treated with 0.1% DMSO or 100 μM CK-666. The spindle in the CK-666 sample is positioned in the *Z* axis of the image. The spindle failed to rotate or form a polar body protrusion. Bar, 50 μm **(C)** Quantification of polar body formation in DMSO or CK-666 samples for three experimental replicates, 100 oocytes scored per condition per experiment (Mean ± SEM, ****p < 0.0001). **(D)** *P. miniata* oocytes co-expressing Lifeact (white), GFP-tubulin (red) and either wild-type (WT) or dominant-negative (T17N) Rac. In contrast to WT Rac-injected oocytes, T17N Rac-expressing oocytes failed in polar body formation after spindles failed to dock at the cortex. Bar, 50 μm. **(E)** Quantification of polar body formation in WT or T17N Rac-expressing oocytes for three experimental replicates, 100 oocytes scored per condition per experiment (Mean ± SEM, ***p < 0.001).

Expression of constitutively active Rac had a strong, inhibitory effect on cytokinesis in sea urchin zygotes (Figure 2), in agreement with studies in cultured cells (Yoshizaki et al., 2004). However, both Rac and Arp2/3 are required for meiotic divisions in mouse oocytes, through the regulation of cytoplasmic streaming and spindle migration (Halet and Carroll, 2007; Leblanc et al., 2011; Sun et al., 2011a; Chaigne et al., 2013, 2016; Yi et al., 2013; Wang et al., 2014). Polar body formation during meiotic maturation is thought to occur through a combination of a SCW, a localized protrusion of the cortex induced by the meiotic spindle, and the canonical cytokinetic signaling apparatus (Maddox et al., 2012; Satoh et al., 2013; Bischof et al., 2017; Klughammer et al., 2018). To investigate the potentially opposing roles of Rac in meiotic cytokinesis, *Patiria miniata* oocytes expressing markers for actin, Rho activity and the Arp2/3 complex were imaged during first meiosis. Polar body extrusion begins at the vegetal pole with a SCW that traverses the egg and terminates at the site of polar body extrusion, where the centralspindlin complex organizes a contractile ring (Figure 4A and Supplementary Movies S5−S7). This wave could be easily observed with either Lifeact or rGBD-GFP, a rhotekin-based biosensor that labels zones of active Rho (Benink and Bement, 2005). In contrast to these probes as well as the formin Diaphanous (Ucar et al., 2013) that label the constricting neck of the polar body protrusion, the Arp2/3 subunit ArpC1 only weakly labeled the cortex until the initiation of polar body extrusion, when it could be observed at the cortex in association with the protruding spindle pole (Figure 4A, arrowhead and Supplementary Movie S7). The meiotic spindles of sea star oocytes form near the site of polar body extrusion, and in contrast to controls, Arp2/3 inhibition resulted a failure in the spindle to induce a protrusion and polar body formation failed (Figures 4B,C and Supplementary Movie S8). Of the 15 cells observed by time-lapse microscopy, five oocytes displayed spindles that failed to dock at the cortex; and of the other 10 samples, eight oocytes failed to form a protrusion and two were able to form a shallow protrusion which was absorbed back into the cytoplasm. Similar results were observed in oocytes expressing dominant-negative (T17N) Rac (Figures 4D,E) but in most cases (10 of 12 oocytes), the spindle failed to dock at the animal cortex, and instead drifted deep into the cytoplasm (Supplementary Movie S9). Thus, in contrast to the mitotic divisions in the sea urchin embryo, Rac and Arp2/3 were required for proper spindle docking and polar body extrusion in sea star oocytes.

**FIGURE 5:**
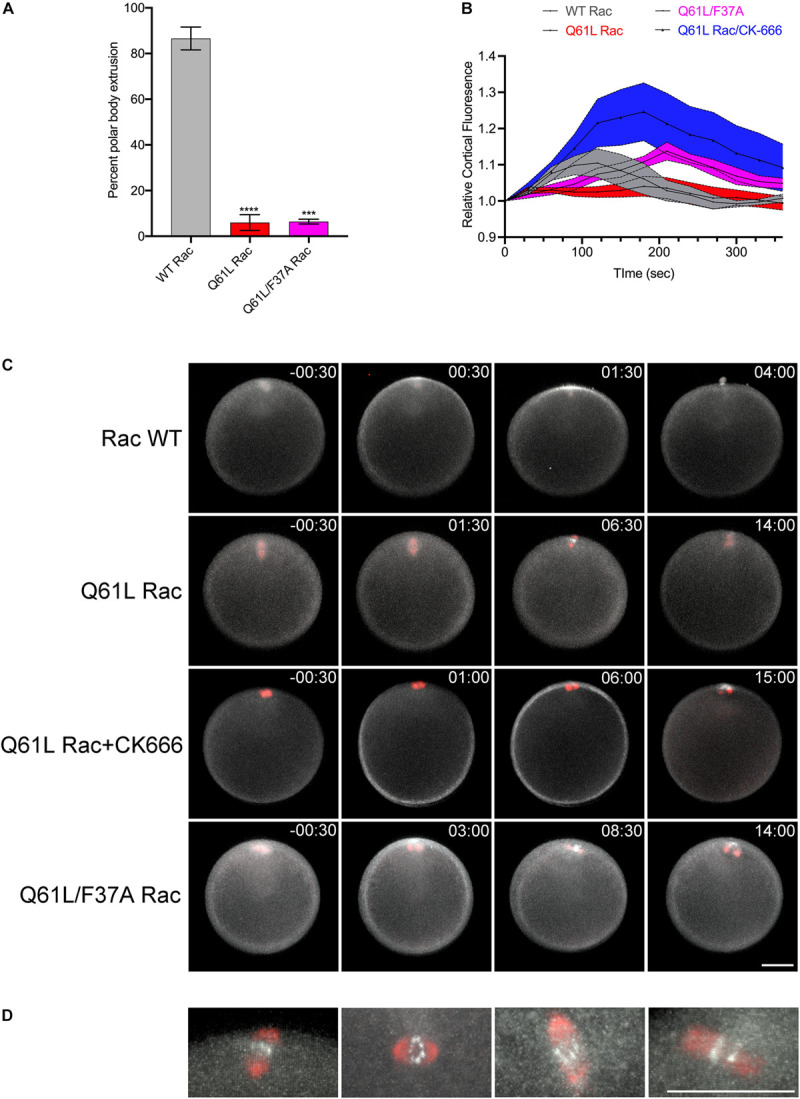
Rac and Arp2/3 directly antagonizes Rho activation during meiosis I. **(A)** Quantification of polar body formation in *P. miniata* oocytes injected WT, Q61L or Q61L/F37A Rac (final intracellular concentration 1.8 μg/ml), 200 oocytes scored per condition per experiment (Mean ± SEM, ***p < 0.001; ****p < 0.0001). **(B)** Cortical Rho activity during the surface contraction wave (SCW) in oocytes expressing WT Rac (gray), Q61L Rac (red), Q61L/F37A Rac (magenta) or Q61L Rac oocytes treated with 100 μM CK-666 (blue). Rhotekin-GFP fluorescence was measured for the entire cortex, where time 0 denotes the initiation of the SCW at the vegetal pole. Mean ± SEM, 7 oocytes per condition. **(C)** SCW and polar body extrusion in *P. miniata* oocytes co-expressing rGBD-GFP (white), mCherry-EMTB (red) and Rac variants described in panel **(B)**. Time point indicate minutes post-initiation of the SCW. Bar, 50 μm. **(D)** Examples of Rho activity associated with meiotic spindles after cytokinesis failure in Q61L Rac-expressing cells. The first panel is the same cell shown in panel **(B)**. Bar, 50 μm.

Expression of constitutively active Rac suppressed polar body extrusion without affecting spindle migration or rotation (Figures 5A,C and Supplementary Movie S10). Interestingly, active Rac did not block Rho activity associated with the central spindle in oocytes that failed to form a protrusion, with bright foci of rGBD-GFP observed at the spindle midzone of anaphase I spindles (Figures 5C,D). Polar body extrusion also failed in the Q61L/F37A mutant (Figures 5A,C and Supplementary Movie S11), possibly due to a dominant-negative effect that has been reported for this mutation (Schwartz et al., 1998). As mentioned above, polar body extrusion initiates with a Rho-dependent SCW triggered at the vegetal pole (Bement et al., 2015; Bischof et al., 2017), and quantification of cortical Rho activity revealed that the Rho wave observed in controls was suppressed in Q61L Rac-expressing oocytes (Figures 5B,C and Supplementary Movie S10). However, the wave of Rho activity associated with the SCW could be rescued in cells expressing Q61L/F37A Rac, albeit with a delay (Figures 5B,C and Supplementary Movie S11). Further, treatment of Q61L Rac-expressing oocytes with CK-666 dramatically enhanced the Rho wave relative to oocytes expressing WT Rac alone (Figures 5B,C and Supplementary Movie S12). This enhancement of cortical Rho activity could be replicated in oocytes treated with CK-666 in the absence of ectopic Rac (Supplementary Figures S2E−I and Supplementary Movie S13), suggesting that inhibition of branched actin networks may affect the amplitude and dynamics of Rho activation in these cells.

## Discussion

There is now a general consensus that Rappaport’s “cleavage stimulus” involves the Chromosomal Passenger, PRC1/Kif4A, and centralspindlin complexes that organize the post-anaphase central spindle and direct the local activation of Rho (Glotzer, 2009; Mishima, 2016; Basant and Glotzer, 2018). The notion of negative regulatory cues have a long history (Wolpert, 1960) and there is evidence supporting the idea that there are antagonistic signals or mechanisms for clearing contractile proteins from the polar regions (Murthy and Wadsworth, 2008; Kunda et al., 2012; Rodrigues et al., 2015; Mangal et al., 2018; Chapa-Y-Lazo et al., 2020). Rac has also been implicated as a negative regulator for cytokinesis (D’Avino et al., 2004; Yoshizaki et al., 2004; Canman et al., 2008; Cannet et al., 2014), with proposed downstream effectors including PAK-mediated cell adhesion (Bastos et al., 2012) and the Arp2/3 complex (Canman et al., 2008). In this report, we explored Rac and Arp2/3 function in sea urchin zygotes, where these factors are dispensable for cytokinesis as well as in sea star oocytes, where both factors are required for polar body extrusion. In both cases, dysregulated Rac blocked cytokinesis in an Arp2/3-dependent manner, and in the case of the SCW, Arp2/3 directly suppresses Rho activity. Together, these results suggest a novel mechanism by which branched actin networks may act as a break against Rho-dependent contractility, which may be applicable not only to canonical cleavages, but to other cell polarization events as well.

### Rac and Cytokinesis in the Sea Urchin Zygote

Considered a “classic” model for studying cell division, echinoid embryos continue to provide mechanistic insights into cytokinesis (Mabuchi et al., 1993; Bement et al., 2005; Foe and von Dassow, 2008; von Dassow et al., 2009; Argiros et al., 2012; Su et al., 2014; Henson et al., 2017). A baseline characterization of actin dynamics revealed a series of dynamic changes in actin-based structures that occur throughout mitosis and cytokinesis, including dramatic changes in deep cytoplasmic actin, cortical actin, and microvilli (Figure 1 and Supplementary Movie S1). However, of the actin populations visible by diffraction-limited imaging, only the global increase in cortical actin preceding furrow initiation was Rho-dependent (Figures 1H,K,M and Supplementary Movie S2), consistent with prior observations of Rho-dependent increases in cortical stiffness accompanying the metaphase-anaphase transition in these cells (Lucero et al., 2006).

**FIGURE 6:**
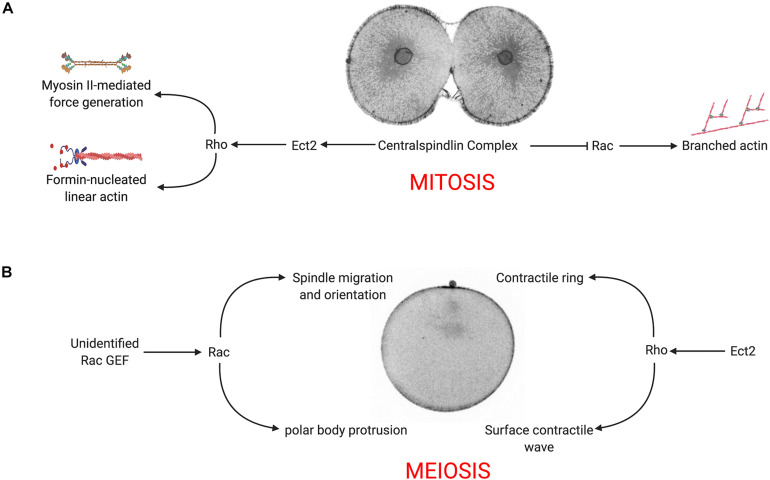
Model for Rac-Arp2/3 signaling during mitotic and meiotic divisions. **(A)** In the presence of WT Rac, the centralspindlin complex recruits Ect2 to promote equatorial Rho activity, which in turn stimulates formin-nucleated actin, myosin II activation and contractile ring assembly. Simultaneously, centralspindlin blocks Rac and Arp2/3 activity, downregulating branched actin networks at the cell equator. **(B)** During meiosis, Rho is activated at the vegetal pole, inducing a surface contractile wave that converges on the animal pole where the centralspindlin complex organizes the ring that completes cytokinesis and polar body extrusion. Rac (activated by an unidentified exchange factor) promotes Arp2/3-mediated branched actin that is required for proper positioning of the spindle and polar body protrusion.

Constitutively active Rac had a strong, negative effect on cytokinesis (Figures 2A−C and Supplementary Movie S3), and suppressed cytoplasmic actin, microvillar and cortical actin dynamics (Figures 2E,F). Normal actin dynamics and cytokinesis were rescued if constitutively active Rac contained second, effector-binding domain that blocks WAVE activation (Figures 2A,E,F and Supplementary Movie S3), suggesting that Rac’s effects on cell division were due its downstream effector, Arp2/3. Arp2/3 is progressively cleared from the cell equator in dividing embryos (Figures 3A−B), and inhibition of Arp2/3 slows but does not inhibit cytokinesis (Figures 3C,D,G). While Arp2/3 was no essential for furrow formation, direct inhibition of Arp2/3 in Q61L-expressing embryos rescued both cytokinesis as well as cortical thickening (Figures 3G,I), suggesting that Arp2/3 was antagonizing Rho-dependent processes. In *C. elegans*, it has been proposed that Rac-activated Arp2/3 might compete with formins for actin monomers (Canman et al., 2008). However, at the level of resolution used for these live cell imaging experiments, it is not clear that activated Rac was significantly shifting the equilibrium between branched and unbranched actin populations (Figures 2E,F, 3E,F). Together, our findings that deregulated activated Rac strongly suppresses cytokinesis in an Arp2/3-dependent manner, supporting the notion that under control conditions, centralspindlin promotes equatorial ring assembly while suppressing Rac and branched actin assembly (Figure 6A; Canman et al., 2008; Cannet et al., 2014; Zhuravlev et al., 2017). Further exploration of the relationship between Rho and Arp2/3 in other cell types will reveal whether this is a general phenomenon.

### Role of Rac and Arp2/3 During Polar Body Extrusion

In addition to representing a highly asymmetric cell division, polar body formation differs from conventional cell divisions in that the Rho signal originates not from the central spindle, but at opposite end of the oocyte, where the initiation of CDK1 inactivation triggers a Rho-dependent SCW (Bischof et al., 2017). Coupled with a protrusion induced by the meiotic spindle, the SCW converges at the animal pole, where central spindle signaling organizes the contractile ring to pinch off the polar body. Polar body formation also differs from mitotic cleavages in its requirement for Rac and Arp2/3. The protrusion of the polar body is thought to be a function of Cdc42 (Zhang et al., 2008), but we and others have shown that Rac functions in polar body formation (Figure 4A; Halet and Carroll, 2007), and Arp2/3 has been implicated in both spindle migration and polar body formation (Sun et al., 2011a,b; Chaigne et al., 2013; Yi et al., 2013; Wang et al., 2014, 2016). Indeed, Arp2/3 is enriched in the forming polar body (Figure 4A) as well as following polar body extrusion (Toledo-Jacobo et al., 2019). Inhibition of either Rac or Arp2/3 blocks spindle alignment and polar body extrusion (Figures 4B−E), and while many of these failures can be attributed to spindle migration defects, membrane bulge formation still fails even when the spindle is immediately subjacent to the membrane (Figure 4B and Supplementary Movie S8). Thus, under normal conditions, both Rho and Rac play essential roles in polar body formation, where Rho provides contractile force through the SCW and contractile ring, and Rac promotes spindle anchoring to the cortex and promotes bulge formation (Figure 6B).

The SCW provided an ability to examine Rac and Rho antagonism independently of the central spindle. In oocytes, activated Rac blocked polar body formation, but also depressed the Rho wave (Figures 5A−C), raising the possibility that active Rac could block polar body extrusion by suppressing the Rho-dependent SCW, consistent with the models for polar body extrusion where cortical contractility is coupled with protrusive activity (Zhang et al., 2008; Satoh et al., 2013). Although polar body extrusion was not rescued under these conditions, both the F37A effector-binding mutation and the Arp2/3 inhibitor rescued the Rho wave in the presence of activated Rac (Figures 5B,C), and in the case of direct Arp2/3 inhibition, the amplitude and persistence of the Rho wave was dramatically increased (Figures 5A−C and Supplementary Movie S12). Further, Arp2/3 inhibition alone increased the amplitude and duration of the Rho wave (Supplementary Figure S2 and Supplementary Movie S13), arguing that Rac indirectly antagonizes Rho through Arp2/3. The notion that actin networks may downregulate Rho has been observed in these same cells, where pulsatile waves of Rho and actin induced by Ect2 overexpression are propagated through a negative feedback loop (Bement et al., 2015). Given that Ect2 is thought to be the exchange factor involved in Rho waves in sea star oocytes (Bement et al., 2015), it is possible then that Arp2/3 nucleated actin may directly interfere with Ect2 activity.

Arp2/3 may modulate contractility by regulating Rho activity, but these experiments do not address the possibility that branched actin networks may act more directly to serve as a break for myosin contractility. There is an abundance of evidence demonstrating that crosslinking proteins tune actomyosin contractility during cytokinesis (Girard et al., 2004; Zhang and Robinson, 2005; Mukhina et al., 2007; Ennomani et al., 2016; Ding et al., 2017; Descovich et al., 2018). In reconstituted systems, disordered networks generated by the inclusion of Arp2/3 are less contractile than ordered bundles (Ennomani et al., 2016), and in living cells, Arp2/3 has been shown to restrict myosin II contractility in growth cones (Yang et al., 2012). More recently, it has been demonstrated that in mollusk embryos, Arp2/3 defines the domain of vegetal cortex that is sequestered by a the polar lobe, a transient constriction that forms at an angle normal to the cleavage plane (Toledo-Jacobo et al., 2019). Thus, regardless of the exact mechanism by which Arp2/3 affects Rho-mediated contractility, branched actin networks may represent an additional mechanism by which cells may spatially control contractility during cell shape change.

## Materials and Methods

### Procurement and Culture of Echinoderm Gametes and Embryos

*Lytechinus pictus* and *S. purpuratus* sea urchins were obtained from Marinus Scientific and Point Loma Marine, and were maintained in a chilled saltwater aquarium at 11–14°C. The sea urchin *L. variegatus* was obtained from Reeftopia and maintained at 20°C. Gametes were obtained by intracoelomic injection of 0.5 M of KCl, and eggs were collected by inverting the urchin over a small beaker of Artificial Sea Water (ASW) while the sperm was collected dry and used immediately or stored at 4°C. Eggs were fertilized with a 1:10,000 dilution of sperm in artificial seawater containing 1 mM 3-amino-triazole, and fertilization envelopes were then removed by passing embryos through Nitex mesh. Embryos were then cultured at 14°C for *S. purpuratus* and *L. pictus* and 20°C for *L. variegatus* until use.

The bat star *P. miniata* was obtained from Marinus and kept in a chilled aquarium as described above. To obtain fully grown, immature oocytes, an incision was made on the ventral surface of an arm, and a small amount of ovary tissue was removed and washed twice in chilled calcium-free artificial sea water (CaFASW) for 15 min to remove follicle cells. Ovary tissue was then placed in 10 μM acetylcholine-ASW for 8–10 min to promote extrusion of oocytes. Oocytes were then placed in fresh ASW containing 100 μg/ml gentamycin, where they could be cultured at 14°C for 3–5 days without any loss of viability. Oocyte maturation was induced by addition of 1–2 μM 1-methyladenine (Acros #AC201310250), where germinal vesicle breakdown (GVBD) occurred after 20–30 min, and first polar body extrusion occurred about 90 min post-hormone addition.

### Reagents and Small Molecule Inhibitors

Unless otherwise specified, all chemicals and reagents were purchased from Sigma. C3 transferase was obtained from Cytoskeleton (#CT03), The Arp2/3 inhibitor CK-666 was purchased from Tocris (#3950), resuspended in Dimethyl Sulfoxide at a stock concentration of 100 mM, and aliquoted stocks stored at −80°C.

### Recombinant Proteins

Recombinant Lifeact-EGFP (Gift from David Burgess, Boston College) was expressed in BL21(DE3) STAR cells (Thermo Fisher Scientific #C601003) and recombinant protein expression was induced for 6 h with 1 mM IPTG. Cells were harvested by centrifugation and protein purified using a Qiagen Ni NTA kit (#30600). Protein fractions were then dialyzed in Phosphate-Buffered Saline (PBS) + 10% glycerol overnight at 4°C, snap frozen in liquid nitrogen and stored at −80°C.

### Immunofluorescence

To visualize Arp2/3 in dividing sea urchin embryos, embryos were fixed in Millonig’s fixative (200 mM NaH_2_PO_4_, 136 NaCl, pH 7.0, 3.7% Formaldehyde) and processed for immunolocalization as previously described (Argiros et al., 2012). Arp2/3 was labeled using a mouse anti-Arp3 monoclonal antibody (Sigma, #A5979) diluted 1:100; and affinity purified rabbit anti-sea urchin MKLP1 (Argiros et al., 2012) was used at a dilution of 1:100. Microtubules were labeled with 1:1000 rat anti-tubulin (YL1/2; Santa Cruz Biotechnology, #sc-53029), and primary antibodies were detected using Alexa Fluor-labeled secondary antibodies (Thermo Fisher Scientific # A-21441, A-11031, and A-21247). Hoescht 33342 (Thermo Fisher Scientific # 62249) was included in the secondary antibody to label DNA.

### Molecular Cloning and Mutagenesis

Unless specified otherwise, all molecular reagents were purchased from New England Biolabs, and PCR primers from Integrated DNA Technologies (IDT). The open reading frames for *S. purpuratus* and *P. miniata* Rac were amplified from either random-primed cDNA (*S. purpuratus*) or a synthetic Gblock (*P. miniata*) and subcloned into XhoI-linearized pCS2P+ (gift from Marc Kirschner Addgene, plasmid # 17095) using In-Fusion cloning (Takara, #638912). Constitutively active (Q61L), dominant-negative (T17N), and effector-binding SpRac mutant (F37A) were generated using the QuickChange II XL site-directed mutagenesis kit (Agilent, #200521) in accordance to the manufacturer’s specifications.

To confirm the effects of Rac mutations on actin organization, *S. purpuratus* WT and mutant Rac was subcloned into pLAGFP2A, a pCS2P+ derivative where GFP-lifeact and Rac are expressed as a single open reading frame separated by a 21-residue viral 2A peptide, which upon translation, results in two separate polypeptides (Sepúlveda-Ramírez et al., 2018). Constructs were transfected into human Retinal Pigmented Epithelial (RPE1) cells using Lipofectamine (Life Technologies, #L3000001), and serum-starved overnight to reduce serum-stimulated actin polymerization. Prior to fixation, cells were treated with SIR-actin (Cytoskeleton, #CY-SC001) for 30 min. Coverslips were then fixed and processed for fluorescence imaging as previously described (Shrestha et al., 2012).

### *In vitro* Transcription

The following pCS2-based constructs: Echinoderm WT and Rac mutants, EMTB-mCherry (gift from William Bement, Addgene # 26742), GFP-rGBD (gift from William Bement, Addgene # 26732), and pLAGFP2A-GFP-Tubulin (Sepúlveda-Ramírez et al., 2019) were linearized with Not I, and capped, poly-adenylated mRNAs synthesized *in vitro* using the SP6 mMessage mMachine and Poly(A) tailing kits (Thermo Fisher Scientific, #AM1340, AM1350). RNA was precipitated with Lithium chloride, suspended in nuclease-free water, quantified by spectrophotometry and stored at −80°C. Lifeact-GFP in pSP64T (gift from Mamiko Yajima, Brown University) was linearized with SalI and transcribed as detailed above. ArpC1 in pGEMHE (gift from Peter Lenart), (Max Planck Institute for Biophysical Chemistry) was *in vitro* transcribed with T7 mMessage mMachine (Thermo Fisher Scientific # AM1344) and poly-adenylated and precipitated as described above.

### Microinjection

Sea urchin eggs were fertilized, stripped of fertilization envelopes, and then transferred to a 35-mm glass bottom culture dish (World Precision Instruments, #FD35). Cells were microinjected with glass capillary micropipettes (World Precision Instruments, #TW100F-3) backfilled with *in vitro* transcribed RNA (500 μg/ml for short term experiments in sea urchin embryos, 300 μg/ml for oocytes). For experiments in sea urchins, recombinant Lifeact-EGFP was co-injected to label the actin cytoskeleton. *P. miniata* oocytes were injected in a 35 mm culture dish with 180 μm Nitex mesh adhered to bottom of the dish to immobilize the oocytes. All injections were carried out on a Zeiss Axiovert 200 M inverted microscope with a Brook temperature stage set to 14°C, using a Parker-Hannifin Picospritzer II pressure injection system with Leica M micromanipulators. Embryos were either imaged immediately (for the first mitotic division) or cultured overnight at 14°C.

### Image Acquisition and Processing

Wide-field epi-fluorescent and DIC time-lapse imaging was performed on a Zeiss Axiovert 200 M inverted microscope equipped with standard epi-fluorescence and Apotome structured illumination imaging, Leica M and Narishige micromanipulators and a Brook temperature-controlled stage. Images were obtained with an AxioCam MRm CCD camera driven by AxioVision 4.8 software.

Sea urchin embryos were imaged using a Leica TCS SP5 II confocal microscope driven by Leica Application Suite software. 512 × 512 images were acquired in resonant scanning mode, which allowed for rapid scanning and averaging. *P. miniata* oocytes were imaged using an Andor Dragonfly 505 spinning disk confocal system mounted on an Olympus IX83 inverted microscope equipped with an Oko Touch microscope stage incubator set to 14°C. 20 μm slabs (40 images at 0.5 μm intervals) were acquired every 30 s using a 60× Aprochromat silicon objective (NA 1.30) and an Andor iXon 888 EMCCD camera driven by Andor Fusion software. Images were compiled and processed using Fusion and Imaris (v9.0.2) software.

### Image Analysis

Image stack manipulations, maximum intensity projections and kymographs were generated using FIJI (ImageJ, National Institutes of Health). To measure the relative cortical distribution of Arp2/3 in fixed sea urchin embryos, fluorescence intensity measurements were made of cross section images using a 45 × 8 pixel ROI for each polar and equatorial surface and a polar: equatorial ratio was calculated per cell for a total of 20 cells were measured per stage. Changes in cortical thickness and microvillar length were measured using ImageJ, taking measurements prior to the metaphase-anaphase transition, and then just prior to the normal timing of furrow initiation at 4 locations on the cortex and the fold increase of thickness was calculated and averaged for each cell. To quantify changes in cytoplasmic actin during mitosis and cortical Rho activity during meiosis, the cytoplasmic fluorescence intensity of Lifeact-GFP and the cortical fluorescence intensity of the Rhotekin biosensor (GFP-rGBD) were measured in time-lapse sequences using the QuimP plugin for ImageJ (Piotr et al., 2018). The resulting fluorescence intensity values for cytoplasmic actin was then normalized to the image frame corresponding to 1 min past NEB. For cortical Rho activity, fluorescence intensity was normalized to the image frame 30 s before the first detection of the surface contractile wave.

### Statistical Analysis

Statistical significance was determined using paired one or two-way Analysis of Variance (ANOVA) tests followed by Tukey-Kramer *post hoc* test with a 95% confidence interval. For frequency data, data were arcsin-square root transformed followed by two-way ANOVA and a Tukey-Kramer *post hoc* test. *T*-tests were used for pairwise comparisons, with a 95% confidence level. All statistics and graphs were generated using GraphPad Prism 8.

## Data Availability Statement

The raw data supporting the conclusion of this article will be made available by the authors, without undue reservation, to any qualified researcher.

## Author Contributions

CS, JH, DP, and AE contributed to the conceptualization and development of the project. AE, DP, SS-R, TS, RD, IT, and GR all contributed to the experimentation and data analysis. IT, GR, AE, DP, and SS-R all contributed to the figure preparation and visualization. CS, JH, AE, GR, SS-R, and DP contributed to the writing and editing of the manuscript. CS and JH secured funding. All authors contributed to the article and approved the submitted version.

## Conflict of Interest

The authors declare that the research was conducted in the absence of any commercial or financial relationships that could be construed as a potential conflict of interest.

## Supplementary Material

Supplementary Figure S1 Characterization of sea urchin Rac mutants. **(A)** Sequence alignment of *Strongylocentrotus purpuratus* (SpRac), *Lytechinus variegatus* (LvRac), *P. miniata* (PmRac) and *Homo sapiens* Rac1. Positions of dominant-negative (T17N), constitutively active (Q61) and effector binding domain (F37) mutants are highlighted in yellow, gray and cyan, respectively. **(B)** Rac is dispensable for early sea urchin development. WT or T17N Rac was injected into fertilized *S. purpuratus* eggs, and embryos were scored for developmental defects at 24 h post-fertilization, when the embryos are normally at the mesenchyme blastula stage **(panel a)**. With increasing amounts of injected RNA, a range of phenotypes were observed such as **(panel b)** a lack of epithelial-mesenchymal transition by the primary mesenchyme cells (PMCs); **(panel c)** embryos with dispersed and dying PMCs; **(panel d)** a lack of blastocoel expansion; and **(panel e)** embryos that did not develop past the cleavage stage. Although dose-dependent trends could be seen (particularly in blastulae containing disorganized PMCs in the blastocoel, orange), there was no significant difference in viability between WT and T17N Rac expressing embryos. Mean ± SEM for a minimum of three experimental replicates, with >24 cells per experiment. ^∗∗∗^*p* < 0.001. **(C)** Ectopic expression of sea urchin Rac in serum-starved hTERT-immortalized RPE1 cells. Transfected cells are marked by Lifeact-GFP, with all cells highlighted with SIR-actin (white) and Hoescht 33342 (blue). Note that presence of lamellipodia in serum-starved, Q61L Rac expressing cells **(C, panels d–f)**. Bar, 25 μm.

Supplementary Figure S2 Arp2/3 inhibition amplifies the Rho wave in sea star oocytes. **(A–H)** Surface contraction wave (SCW) and polar body extrusion in *P. miniata* oocytes co-expressing rGBD-GFP (white) and mCherry-EMTB (red) and treated with either 0.1% DMSO **(A–D)** or 100 μM CK-666 **(E–H)**. Bar, 50 μm. Whereas the Rho activity in DMSO controls traversed as a wave and terminated with polar body extrusion, CK-666 treated oocytes exhibited a delayed and dramatically elevated Rho wave. **(I)** Rhotekin-GFP fluorescence was measured for the entire cortex in DMSO and CK-666 treated oocytes, where time 0 denotes the initiation of the SCW at the vegetal pole. Mean ± SEM, 7 oocytes per condition.

Supplementary Movie S1 Actin dynamics during the first mitotic division the sea urchin embryo. Single plane time-lapse movie of a *L. pictus* embryo injected with recombinant Lifeact-GFP. Images were acquired every 20 s and played back at 10 frames/second.

Supplementary Movie S2 C3 Transferase blocks cytokinesis without affecting cytoplasmic or microvillar actin dynamics. Single plane time-lapse movie of a *L. pictus* embryo co-injected with recombinant Lifeact-GFP and C3 transferase. Images were acquired every 30 s and played back at 10 frames/second.

Supplementary Movie S3 Actin dynamics and cytokinesis in embryos expressing wild-type or mutant Rac. *L. variegatus* embryos were co-injected with Lifeact-GFP and either WT, Q61L, or Q61L/F37A Rac mRNA and imaged during the first division. Images were acquired every 30 s and played back at 10 frames/second.

Supplementary Movie S4 Aberrant actin dynamics in a multinucleate embryo expressing constitutively active Rac. *L. variegatus* embryos were co-injected with Q61L Rac mRNA and recombinant Lifeact-GFP and imaged during the third division. Images were acquired every 30 s and played back at 10 frames/second.

Supplementary Movie S5 Actin dynamics during the first polar body formation. Time-lapse movie of a *P. miniata* oocyte injected with bicistronic mCherry-Lifeact-GFP tubulin mRNA RNA to simultaneously image actin (white) and microtubules (red). A 20 μm surface image was acquired at 0.5 μm intervals every 60 s and played back at 5 frames/second.

Supplementary Movie S6 Active Rho dynamics during the first polar body formation. Time-lapse movie of a *P. miniata* oocyte injected with rGBD-GFP and mCherry-ETMB mRNA to image active Rho (white) and microtubules (red). A 20 μm medial slab was acquired at 0.5 μm intervals every 30 s and played back at 10 frames/second.

Supplementary Movie S7 Arp2/3 dynamics during the first polar body formation. Time-lapse movie of a *P. miniata* oocyte, co-injected with ArpC1-GFP and mchETMB mRNA to image the Arp2/3 complex (white) and microtubules (red). A 20 μm medial slab was acquired at 0.5 μm intervals every 30 s and played back at 10 frames/second.

Supplementary Movie S8 Polar body extrusion fails in presence of the Arp2/3 inhibitor CK-666. Time-lapse movie of a *P. miniata* oocyte injected with bicistronic mCherry-Lifeact-GFP tubulin mRNA to simultaneously image actin (white) and microtubules (red) in the presence of 0.1% DMSO or 100 μM CK-666. A 20 μm medial slab was acquired at 0.5 μm intervals every 30 s and played back at 10 frames/second.

Supplementary Movie S9 Polar body extrusion fails in an oocyte expressing dominant negative Rac. Time-lapse movie of a *P. miniata* oocyte injected co-injected with either WT Rac or T17N Rac and bicistronic mCherry-Lifeact-GFP tubulin mRNA to simultaneously image actin (white) and microtubules (red). A 20 μm medial slab was acquired at 0.5 μm intervals every 30 s and played back at 10 frames/second.

Supplementary Movie S10 Suppression of the Rho wave and failure of polar body extrusion in an oocyte expressing constitutively active Rac. Time-lapse movie of a *P. miniata* oocyte injected with either WT Rac or Q61L Rac with rGBD-GFP and mCherry-ETMB RNA to simultaneously image active Rho (white) and microtubules (red). A 20 μm medial slab was acquired at 0.5 μm intervals every 30 s and played back at 10 frames/second.

Supplementary Movie S11 Rescue of the Rho wave but failure of polar body extrusion in an oocyte expressing Q61L/F37A Rac. Time-lapse movie of a *P. miniata* oocyte injected with Q61L/F37A Rac, rGBD-GFP and mCherry-ETMB RNA to simultaneously image active Rho (white) and microtubules (red) A 20 μm medial slab was acquired at 0.5 μm intervals every 30 s and played back at 10 frames/second.

Supplementary Movie S12 Arp2/3 inhibition rescues the Rho wave but not polar body extrusion in an oocyte expressing constitutively active Rac. Time-lapse movie of a *P. miniata* oocyte injected with Q61L Rac, rGBD-GFP and mCherry-ETMB RNA to simultaneously image active Rho (white) and microtubules (red) in presence of 100 μM CK-666. Images were acquired every 30 s and played back at 10 frames/second. A 20 μm medial slab was acquired at 0.5 μm intervals every 30 s and played back at 10 frames/second.

Supplementary Movie S13 Arp2/3 inhibition enhances the Rho wave during surface contraction during polar body extrusion. Time-lapse movie of a *P. miniata* oocyte injected with rGBD-GFP and mCherry-ETMB RNA to simultaneously image active Rho (white) and microtubules (red) in presence of either 0.1% DMSO or 100 μM CK-666. A 20 μm medial slab was acquired at 0.5 μm intervals every 30 s and played back at 10 frames/second.
