# Supplementary figures and images for: Rac and Arp2/3-Nucleated Actin Networks Antagonize Rho During Mitotic and Meiotic Cleavages
Source: Front Cell Dev Biol. 2020 Nov 17;8:591141. doi: 10.3389/fcell.2020.591141 (PMC7705106; doi:10.3389/fcell.2020.591141)

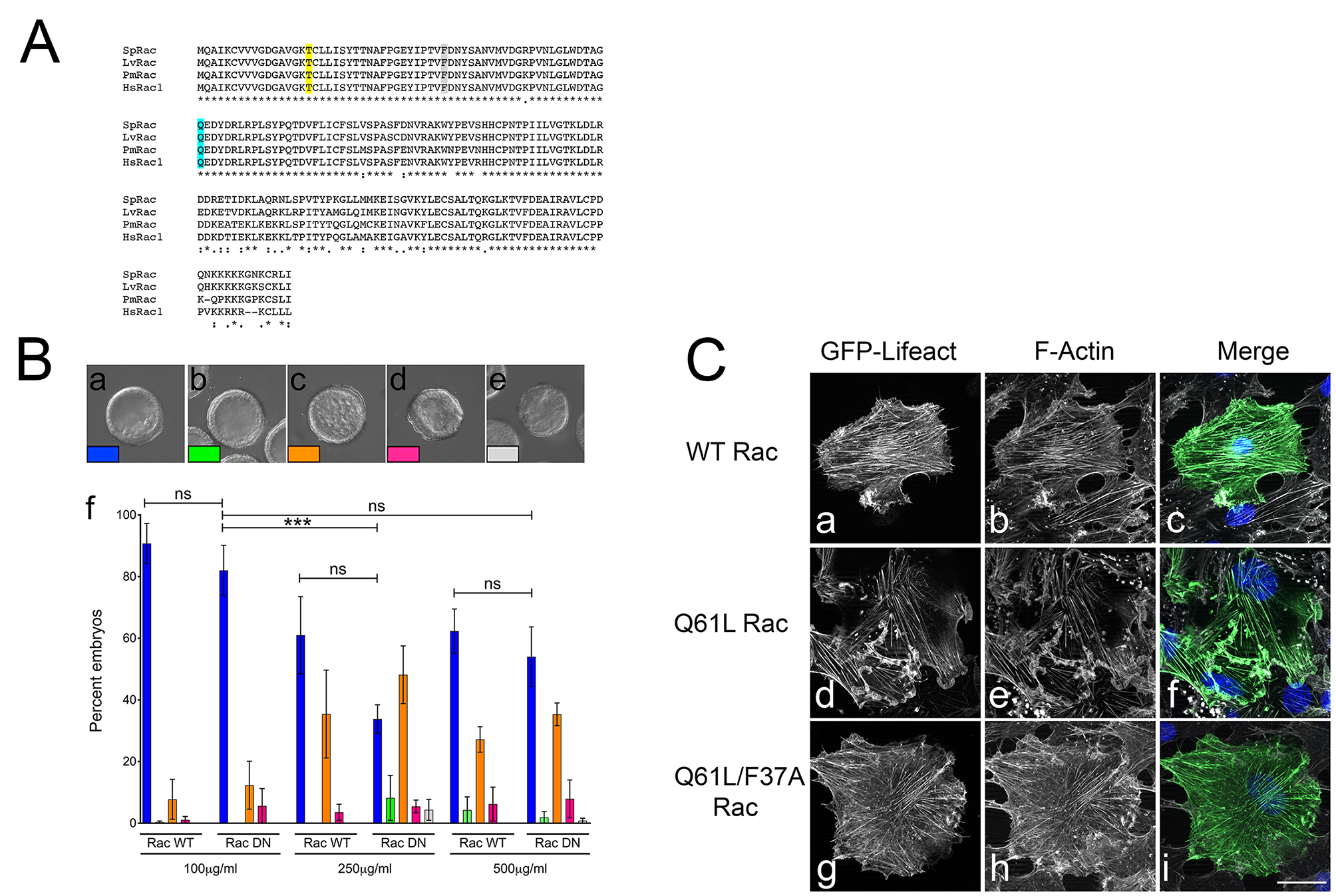

Supplement: Supplementary Figure S1 — Characterization of sea urchin Rac mutants. (A) Sequence alignment of Strongylocentrotus purpuratus (SpRac), Lytechinus variegatus (LvRac), P. miniata (PmRac) and Homo sapiens Rac1. Positions of dominant-negative (T17N), constitutively active (Q61) and effector binding domain (F37) mutants are highlighted in yellow, gray and cyan, respectively. (B) Rac is dispensable for early sea urchin development. WT or T17N Rac was injected into fertilized S. purpuratus eggs, and embryos were scored for developmental defects at 24 h post-fertilization, when the embryos are normally at the mesenchyme blastula stage (panel a). With increasing amounts of injected RNA, a range of phenotypes were observed such as (panel b) a lack of epithelial-mesenchymal transition by the primary mesenchyme cells (PMCs); (panel c) embryos with dispersed and dying PMCs; (panel d) a lack of blastocoel expansion; and (panel e) embryos that did not develop past the cleavage stage. Although dose-dependent trends could be seen (particularly in blastulae containing disorganized PMCs in the blastocoel, orange), there was no significant difference in viability between WT and T17N Rac expressing embryos. Mean ± SEM for a minimum of three experimental replicates, with >24 cells per experiment. ∗∗∗p < 0.001. (C) Ectopic expression of sea urchin Rac in serum-starved hTERT-immortalized RPE1 cells. Transfected cells are marked by Lifeact-GFP, with all cells highlighted with SIR-actin (white) and Hoescht 33342 (blue). Note that presence of lamellipodia in serum-starved, Q61L Rac expressing cells (C, panels d–f). Bar, 25 μm. [file Image_1.TIF]

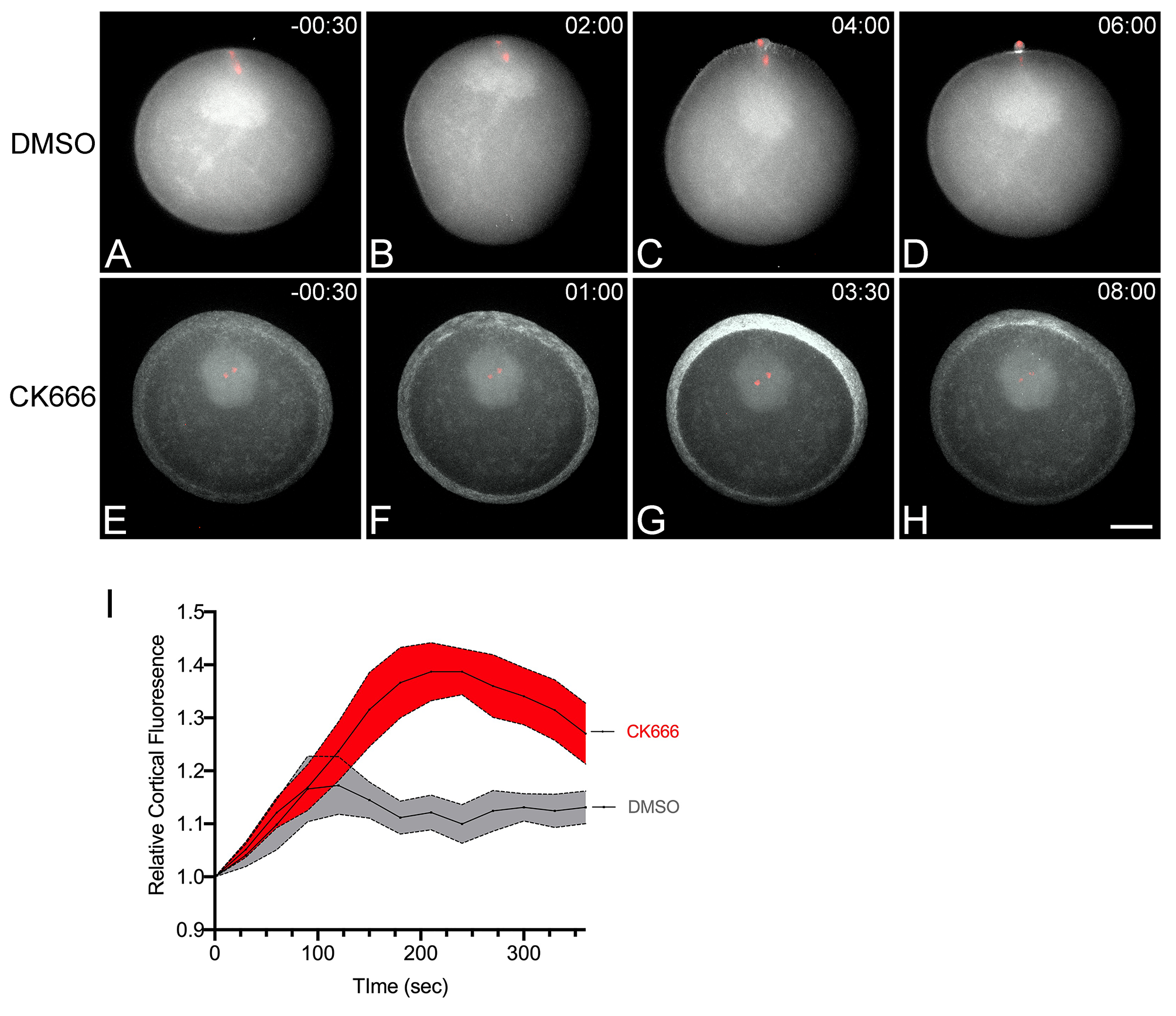

Supplement: Supplementary Figure S2 — Arp2/3 inhibition amplifies the Rho wave in sea star oocytes. (A–H) Surface contraction wave (SCW) and polar body extrusion in P. miniata oocytes co-expressing rGBD-GFP (white) and mCherry-EMTB (red) and treated with either 0.1% DMSO (A–D) or 100 μM CK-666 (E–H). Bar, 50 μm. Whereas the Rho activity in DMSO controls traversed as a wave and terminated with polar body extrusion, CK-666 treated oocytes exhibited a delayed and dramatically elevated Rho wave. (I) Rhotekin-GFP fluorescence was measured for the entire cortex in DMSO and CK-666 treated oocytes, where time 0 denotes the initiation of the SCW at the vegetal pole. Mean ± SEM, 7 oocytes per condition. [file Image_2.TIF]
